# Phage Cocktails with Daptomycin and Ampicillin Eradicates Biofilm-Embedded Multidrug-Resistant *Enterococcus faecium* with Preserved Phage Susceptibility

**DOI:** 10.3390/antibiotics11091175

**Published:** 2022-08-30

**Authors:** Ashlan J. Kunz Coyne, Kyle Stamper, Razieh Kebriaei, Dana J. Holger, Amer El Ghali, Taylor Morrisette, Biswajit Biswas, Melanie Wilson, Michael V. Deschenes, Gregory S. Canfield, Breck A. Duerkop, Cesar A. Arias, Michael J. Rybak

**Affiliations:** 1 Anti-Infective Research Laboratory, College of Pharmacy and Health Sciences, Wayne State University, Detroit, MI 48201, USA; 2 Department of Pharmacy Practice, College of Pharmacy, Nova Southeastern University, Davie, FL 33328, USA; 3 Department of Pharmacy and Clinical Services, College of Pharmacy, Medical University of South Carolina, Charleston, SC 29208, USA; 4 Department of Pharmacy Services, Shawn Jenkins Children’s Hospital, Medical University of South Carolina, Charleston, SC 29208, USA; 5 Naval Medical Research Center, Fort Detrick, MD 21702, USA; 6 Leidos, Reston, VA 20190, USA; 7 Department of Immunology and Microbiology, School of Medicine, University of Colorado, Aurora, CO 80045, USA; 8 Department of Infectious Diseases, School of Medicine, University of Colorado, Aurora, CO 80045, USA; 9 Division of Infectious Diseases, Houston Methodist Hospital, Houston, TX 77030, USA; 10 Center for Infectious Diseases, Houston Methodist Research Institute, Houston, TX 77030, USA; 11 School of Medicine, Wayne State University, Detroit, MI 48201, USA

**Keywords:** *Enterococcus faecium*, bacteriophage cocktails, bacteriophage-antibiotic combinations, daptomycin, beta-lactams, phage-antibiotic synergy

## Abstract

Multidrug-resistant (MDR) *Enterococcus faecium* is a challenging nosocomial pathogen known to colonize medical device surfaces and form biofilms. Bacterio (phages) may constitute an emerging anti-infective option for refractory, biofilm-mediated infections. This study evaluates eight MDR *E. faecium* strains for biofilm production and phage susceptibility against nine phages. Two *E. faecium* strains isolated from patients with bacteremia and identified to be biofilm producers, R497 (daptomycin (DAP)-resistant) and HOU503 (DAP-susceptible dose-dependent (SDD), in addition to four phages with the broadest host ranges (ATCC 113, NV-497, NV-503-01, NV-503-02) were selected for further experiments. Preliminary phage-antibiotic screening was performed with modified checkerboard minimum biofilm inhibitory concentration (MBIC) assays to efficiently screen for bacterial killing and phage-antibiotic synergy (PAS). Data were compared by one-way ANOVA and Tukey (HSD) tests. Time kill analyses (TKA) were performed against R497 and HOU503 with DAP at 0.5× MBIC, ampicillin (AMP) at free peak = 72 µg/mL, and phage at a multiplicity of infection (MOI) of 0.01. In 24 h TKA against R497, phage-antibiotic combinations (PAC) with DAP, AMP, or DAP + AMP combined with 3- or 4-phage cocktails demonstrated significant killing compared to the most effective double combination (ANOVA range of mean differences 2.998 to 3.102 log_10_ colony forming units (CFU)/mL; *p* = 0.011, 2.548 to 2.868 log_10_ colony forming units (CFU)/mL; *p* = 0.023, and 2.006 to 2.329 log_10_ colony forming units (CFU)/mL; *p* = 0.039, respectively), with preserved phage susceptibility identified in regimens with 3-phage cocktails containing NV-497 and the 4-phage cocktail. Against HOU503, AMP combined with any 3- or 4-phage cocktail and DAP + AMP combined with the 3-phage cocktail ATCC 113 + NV-497 + NV-503-01 demonstrated significant PAS and bactericidal activity (ANOVA range of mean differences 2.251 to 2.466 log_10_ colony forming units (CFU)/mL; *p* = 0.044 and 2.119 to 2.350 log_10_ colony forming units (CFU)/mL; *p* = 0.028, respectively), however, only PAC with DAP + AMP maintained phage susceptibility at the end of 24 h TKA. R497 and HOU503 exposure to DAP, AMP, or DAP + AMP in the presence of single phage or phage cocktail resulted in antibiotic resistance stabilization (i.e., no antibiotic MBIC elevation compared to baseline) without identified antibiotic MBIC reversion (i.e., lowering of antibiotic MBIC compared to baseline in DAP-resistant and DAP-SDD isolates) at the end of 24 h TKA. In conclusion, against DAP-resistant R497 and DAP-SDD HOU503 *E. faecium* clinical blood isolates, the use of DAP + AMP combined with 3- and 4-phage cocktails effectively eradicated biofilm-embedded MDR *E. faecium* without altering antibiotic MBIC or phage susceptibility compared to baseline.

## 1. Introduction

Enterococci are challenging nosocomial pathogens known to cause bloodstream and medical device infections (MDI) [1,2,3,4,5,6]. *E. faecium* is infamous for its antimicrobial resistance phenotypes, with greater than 80% of isolates demonstrating vancomycin resistance. Compared to infections caused by vancomycin-susceptible Enterococcus (VSE), infections caused by vancomycin-resistant *E. faecium* (VRE) are of great clinical concern given their association with higher healthcare costs, mortality rates, and longer hospitalizations [7,8]. Critical to the pathogenesis of these infections is the propensity of enterococci to form biofilms, creating a dangerous reservoir of persistent bacteria that readily confers resistance and subsequent morbidity and mortality risk [1,7]. Increasing prevalence of VRE coupled with MDI treatment challenges indicates an urgent need for therapeutic options [9,10].

Daptomycin (DAP) is a preferred treatment for serious VRE infections and has demonstrated rapid biofilm penetration using fluorescent visualization [11,12,13]. Unfortunately, DAP-nonsusceptible (DNS) and DAP-susceptible dose-dependent (SDD) phenotypes are quickly emerging [14,15,16,17,18]. Substitutions in LiaS and LiaR of the LiaFSR pathway, enhances DAP’s selection for resistance, rendering the isolate unresponsive to DAP monotherapy, regardless of dose exposure [19,20]. Notably, strains harboring LiaFSR mutations are also nonresponsive to DAP (8–12 mg/kg/day) and beta-lactam (BL) combinations (DAP-BL) that have synergistic and bactericidal activity against some otherwise nonresponsive VRE strains, even when combinations included DAP 14 mg/kg/day [19,21]. This is extremely problematic in the clinical realm, as most practitioners utilize a “best guess” scenario when choosing which DAP-BL combination to use as first-line therapy or in recalcitrant infections.

Bacteriophages (“phages”) may be an emerging anti-infective treatment option for refractory, MDR, and biofilm-mediated MDI, unresponsive to conventional antibiotics. While successful use has been demonstrated in case reports, efficacy data for phage in clinical trials is lacking and there remains sparse information to guide phage and antibiotic selection in the clinical setting. The decline in antibiotic discovery and emergence of resistance to last line antibiotics, motivates the need for alternative antimicrobials. Given their ability to target specific host bacteria, release infectious progeny on-site, and degrade biofilm matrix exopolysaccharide, phage as an adjunct to antibiotics is increasingly sought after for patients with MDI that are unable to undergo source control due to devastating consequences (e.g., left-ventricular assist device or periprosthetic joint infections) [22,23,24,25,26]. Encouraging interactions have been described with phage-antibiotic combinations (PAC), including synergistic killing of both DAP-resistant and DAP-susceptible dose dependent (SDD) VRE strains in biofilm across multiple PAC, even when DAP-BL combinations fail [27,28]. However, the exquisite selectivity of phage may convey a challenge for its clinical use, namely treatment-emergent phage resistance, secondary to their co-evolution [29,30]. This situation has been demonstrated previously with treatment-emergent phage resistance identified with DAP-BL-single phage combinations against DAP-resistant VRE strains in biofilm [31,32]. The use of phage cocktails may be a useful tool to circumnavigate the potential for bacteria to evolve phage resistance since strains that become resistant to one phage can potentially be targeted by other phages in the cocktail. This observation led us to investigate the hypothesis that PAC with DAP, AMP, and phage cocktails would eradicate biofilms of DAP-resistant and DAP-SDD *E. faecium* while preventing treatment emergent phage resistance.

## 2. Results

### 2.1. Bacterial Isolates

*E. faecium* clinical isolates, R497 and HOU503 were evaluated in this study. R497 is a DAP-resistant (biofilm minimum inhibitory concentration (MBIC) = 16 µg/mL) clinical isolate that harbors the T120S and W73C substitutions in LiaS and LiaR, respectively [33,34]. HOU503 is a vancomycin-resistant (VAN MBIC = 2 µg/mL), DAP-susceptible dose dependent (SDD) (DAP MBIC = 32 µg/mL) clinical strain that harbors T120A and W73C substitutions in LiaS and LiaR, respectively [35,36]. *E. faecium* clinical isolates R497 and HOU503 were selected for additional experiments based on their high biofilm production and phage host range relative to other evaluated *E. faecium* strains, which was evaluated by crystal violet microtiter plate biofilm quantification assay and modified small drop agar method, respectively (Table 1) [37,38,39].

### 2.2. Checkerboard Analyses

**Figure 1:**
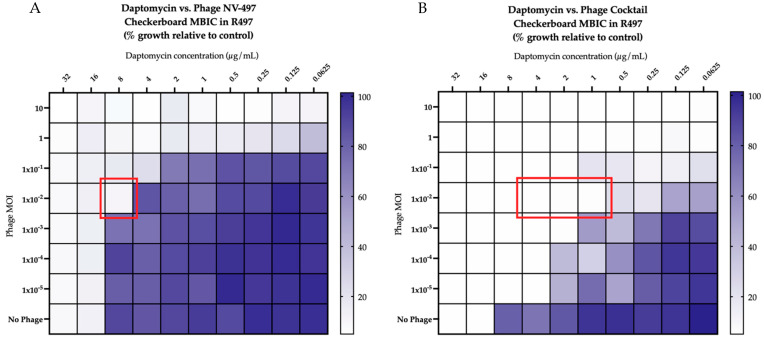
(**A**,**B**). Modified checkerboard minimum biofilm inhibitory concentration (MBIC) analyses of daptomycin combined with phage NV-497 (**A**) and a 4-phage cocktail of ATCC 113 + NV-497 + NV-503-01 + NV-503-02 (**B**) (each phage at MOI 0.01) against DAP-resistant R497. Additivity, defined as an FIC index >0.5 but <4, is indicated by the red outline in (**A**). Synergy, defined as an FIC index ≤0.5, is indicated by the red outline in (**B**). Comparisons are versus growth control and depicted by the purple color gradient as percent of growth.

**Figure 2:**
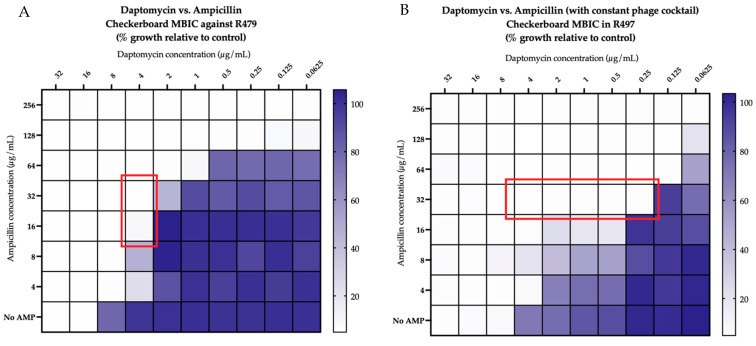
(**A**,**B**). Modified checkerboard minimum biofilm inhibitory concentration (MBIC) analyses of combination daptomycin and ampicillin without (**A**) and a phage cocktail of ATCC 113 + NV-497 + NV-503-01 (**B**) (each at MOI 0.01) against DAP-resistant R497. Synergy, defined as an FIC index ≤0.5, is indicated by the red outline in (**A**,**B**). Comparisons are versus growth control and depicted by the purple color gradient as percent of growth.

Against both R497 and HOU503, DAP in combination with phage NV-497 at a multiplicity of infection (MOI) of 0.01 was additive, with an FIC index of 1 (Figure 1A), while DAP in the presence of a 4-phage cocktail (ATCC 113, NV-497, NV-503-01, NV-503-2; each at an MOI of 0.01), was synergistic, with an FIC index of 0.5 (Figure 1B). The addition of AMP to DAP in the presence of phage NV-497 (MOI 0.01) against DAP-resistant R497 in biofilm was synergistic, with an FIC index of 0.5 (Figure 2A) with additional bacterial killing identified with DAP + AMP in the presence of each 3- and 4-phage cocktail (each at an MOI of 0.01) (Figure 2B not all data shown).

### 2.3. Time Kill Analyses

**Figure 3:**
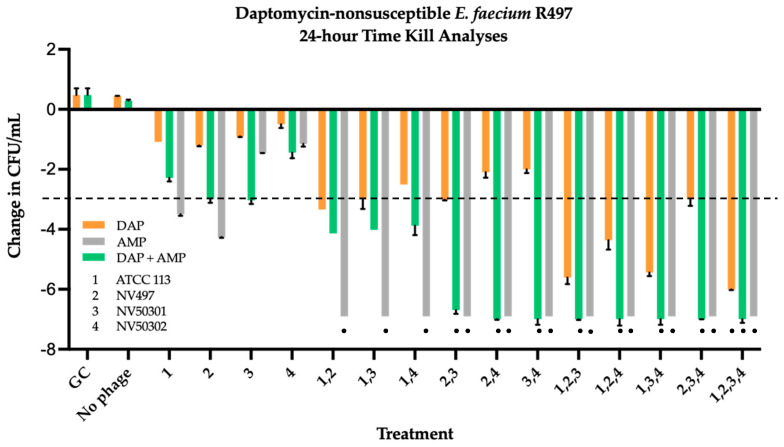
Time kill analyses of DAP at 0.5x MBIC, AMP at free peak = 72 µg/mL, and DAP + AMP, alone and in combination with four bacteriophages, ATCC 113, NV-497, NV-503-01, NV-503-02, each at an MOI of 0.01 against DAP-resistant R497. Change in CFU/mL was in comparison to initial inoculum. Synergy was defined as a ≥2-log_10_-CFU/mL kill compared to the most effective agent (or double-combination regimen) at 24 h (synergistic regimens indicated by black circle). Bactericidal activity was defined as a ≥3-log_10_-CFU/mL reduction from baseline (bactericidal regimens indicated by the bars at or extending below the dashed black line).

**Figure 4:**
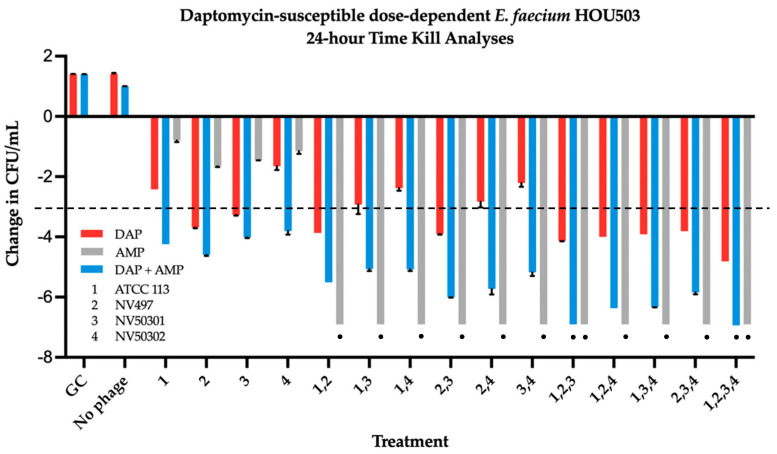
Time kill analyses of DAP at 0.5x MBIC, AMP at free peak = 72 µg/mL, and DAP + AMP, alone and in combination with four bacteriophages, ATCC 113, NV-497, NV-503-01, NV-503-02, each at an MOI of 0.01 against DAP-susceptible dose-dependent HOU503. Change in CFU/mL was in comparison to initial inoculum. Synergy was defined as a ≥2-log_10_-CFU/mL kill compared to the most effective agent (or double-combination regimen) at 24 h (synergistic regimens indicated by black circle). Bactericidal activity was defined as a ≥3-log_10_-CFU/mL reduction from baseline (bactericidal regimens indicated by the bars at or extending below the dashed black line).

DAP-resistant R497 and DAP-SDD HOU503 were evaluated in 24 h biofilm TKA against DAP (0.5× MBIC), AMP (free peak = 72 µg/mL), combination DAP + AMP, single phage (MOI 0.01), phage cocktails (each at an MOI of 0.01), and multiple PAC (Figure 3 and Figure 4). Results from checkerboard analyses were used to determine phage MOI to be used in TKA. Against R497, AMP combined with any 2-, 3-, or 4-phage cocktail demonstrated significant bactericidal and synergistic activity compared to the most effective double combination regimen (ANOVA range of mean differences 2.998 to 3.102 log_10_ colony forming units (CFU)/mL; *p* = 0.011) (Figure 3). This was similar to DAP + AMP PAC, other than those with 2-phage cocktails that contained ATCC 113 Phage (ANOVA range of mean differences 2.548 to 2.868 log_10_ colony forming units (CFU)/mL; *p* = 0.023). PAC with DAP monotherapy required the addition of the 4-phage cocktail to demonstrate bactericidal and synergistic killing (ANOVA range of mean differences 2.006 to 2.329 log_10_ colony forming units (CFU)/mL; *p* = 0.039).

Against DAP-SDD HOU503, AMP combined with each 2-, 3-, or 4-phage cocktail was once again demonstrated significant bactericidal and synergistic activity compared to the most effective double combination, while DAP + AMP required addition of the 3-phage cocktail containing ATCC 113 Phage, NV-497, and NV-503-01, or the 4-phage cocktail for significant bactericidal and synergistic killing (ANOVA range of mean differences 2.251 to 2.466 log_10_ colony forming units (CFU)/mL; *p* = 0.044 and 2.119 to 2.350 log_10_ colony forming units (CFU)/mL; *p* = 0.028, respectively) (Figure 4). DAP monotherapy in PAC, while bactericidal in combination with multiple single and phage cocktail combinations, did not demonstrate synergistic killing against HOU503.

### 2.4. Bacteriophage and Antibiotic Resistance Testing

R497 and HOU503 that survived 24 h TKA involving phage were assessed for phage resistance. Bacteriophage resistance was observed in 24 h R497 TKA samples for each of the four phages in all phage +/− antibiotic regimens except those containing DAP + AMP and NV-497 in 2-, 3-, or 4-phage cocktails (Table 2). In HOU503, phage resistance in TKA samples at 24 h occurred in all combinations of phage plus DAP or AMP, while phage resistance in DAP + AMP combinations was only identified in DAP + AMP combinations that included single phage. Notably, R497 and HOU503 exposure to DAP, AMP, or DAP + AMP in the presence of single phage or phage cocktail resulted in antibiotic resistance stabilization (i.e., no antibiotic MBIC elevation compared to baseline) without identified antibiotic MBIC reversion (i.e., lowering of antibiotic MBIC compared to baseline in DAP-resistant and DAP-SDD isolates) at the end of 24 h TKA.

## 3. Discussion

Recognizing the critical knowledge gap in PAC against VRE, this study aimed to evaluate whether the addition of phage cocktails to SOC antibiotics in PAC demonstrated fundamental PAS components in biofilm state including (i) bacterial eradication; (ii) circumvention of phage:bacterial resistance; and (iii) reversion of resistance (‘resensitization’). Specifically, four different phages (ATCC 113, NV-497, NV-503-01, NV-503-02) were evaluated alone and in combination with DAP, AMP, or DAP + AMP against two clinical vancomycin-resistant *E. faecium* strains isolated from patients with bacteremia, DAP-resistant R497 and DAP-SDD HOU503. Prior to 24 h TKA, modified checkerboard MBICs were used as a preliminary screening method to efficiently evaluate multiple PAC for PAS. Compared to the use of modified checkerboard analyses in planktonic state, which provide information pertaining to the suppression of bacterial growth, modified checkerboards in biofilm state provides additional information related to eradication of biofilm-embedded VRE at 24 h, similar to TKA. The use of modified checkerboard MBIC in this study facilitated accurate and higher throughput screening of effective PAC at various antibiotic concentrations and phage MOI compared to more resource intensive TKA. Results of the modified checkerboard MBIC against R497 and HOU503 were reflective of TKA results with increased bacterial killing identified with DAP combined with a 3-phage cocktail compared to DAP plus single phage. Additional bacterial killing that was then visualized with the addition of AMP to DAP in the presence of a single phage or phage cocktail. These data align with TKA results in which DAP in combination with 3- and 4-phage cocktails demonstrated synergistic activity, however, the addition of AMP achieved killing to detection limit. These data provide validation for the use of a modified checkerboard MBIC to screen for killing effect of standard of care antibiotics and phage cocktails against biofilm-embedded VRE and synergy assessment prior to TKA.

In 24 h biofilm TKA, multiple DAP, AMP, and DAP + AMP combinations that included ≥2 phages in cocktail demonstrated detection level killing at 24 h. However, the prevention of treatment-emergent phage resistance at the end of 24 h TKA required DAP + AMP in combination with 2-, 3- or 4-phage combinations. Similar to previous data in planktonic and biofilm state demonstrating that the VRE strain with high phage susceptibility (R497) showed less emergence of bacteriophage resistance at the end of 24 h TKA compared to the strain with medium phage susceptibility (HOU503), these data demonstrate that while PAC containing DAP, AMP, or DAP + AMP prevented phage resistance at the end of 24 h TKA against R497, against HOU503, DAP + AMP was required in PAC to prevent phage resistance [31]. Regarding antibiotic resistance stabilization, no difference in DAP or AMP MBIC was identified in post-TKA analyses compared to baseline. The observation that phage resistance can be a fitness trade-off under antibiotic pressure in enterococci has been studied to a limited extent previously. In one study by Canfield et al., antimicrobial susceptibility of cell wall and membrane-acting antibiotics, including ampicillin and daptomycin, was tested for phage-resistant *E. faecium* and *E. faecalis* strains harboring mutations in *sagA* and *epa* genes, respectively [40]. They identified that mutations in *sagA* and *epa* genes, but not phage capsule mutations, manifested as enhanced antibiotic susceptibility compared to phage-sensitive strains. Notably, the *epa* gene is associated with teichoic acid biosynthesis so altered teichoic acids at the cell surface of *E. faecalis* may enable enhanced or at least stabilized daptomycin and beta-lactam susceptibility in phage-resistant strains. Further studies are warranted including those with whole genome sequencing and comparative genomics to further evaluate genes important for phage infection of *E. faecium* and antibiotic susceptibility enhancement or stabilization.

Limitations of this study include the assessment of only two MDR *E. faecium* clinical isolates in modified checkerboard MBIC and 24 h TKA. In the context of the current study, the primary goal was to identify which phage cocktails, when combined with standard of care antibiotics, demonstrated bactericidal killing, phage-antibiotic synergy, possible reversion of baseline antibiotic nonsusceptibility, and protection against treatment-emergent phage and antibiotic resistance in 24 h TKA. Additional evaluations of the phage cocktails use in this study against an array of other clinical VRE strains would be highly beneficial in clinical context to identify a phage cocktail with the widest host range. Additionally, based on the dataset generated by this work, there is not a clear formula for the expected outcome when combining these specific phages, and although phage cocktails have been designed and their efficacy reported in the literature previously, guidelines for the design and development of optimized phage cocktails do not exist. Synergistic effects of phage-antibiotic combinations evaluated in this study may be due to specific phage properties not assessed here including (1) adsorption, (2) rate of infection, and (3) progeny production, to name a few. Answering these questions may help determine effective future cocktail design. Furthermore, genotypic analysis of included phages prior to and following their use in 24 h TKA may decipher differences in the emergence of phage resistance at the end of 24 h TKA.

In summary, this study demonstrates that in instances of biofilm-embedded MDR *E. faecium*, the addition of select bacteriophage cocktails to DAP + AMP may be a promising option to eradicate biofilm-mediated infections while preserving baseline antibiotic MBIC and phage susceptibility. Additional complementary studies are warranted to assess phage host range against a larger panel of DAP-resistant and DAP-SDD isolates and evaluation of PAS components including bacterial eradication, circumvention of phage:antibiotic resistance, and reversion of antibiotic and phage resistance over longer time periods in simulated biofilm pharmacokinetic/pharmacodynamic (PK/PD) models to validate the findings of this study. Furthermore, genetic analysis of additional *E. faecium* strains and bacteriophages would provide further insight as to the trends we report here.

## 4. Materials and Methods

### 4.1. Bacterial Isolates

*E. faecium* strains R497 and HOU503, both with LiaFSR mutations and isolated from patients with bacteremia, were selected from a panel of 8 vancomycin-resistant *E.faecium* isolates located in the Anti-Infective Research Laboratory library and evaluated in further experiments [33,34,35,41,42].

### 4.2. Antimicrobial Agents and Media

Antibiotics used in this study (DAP and AMP) were purchased from Sigma Chemical Company (St. Louis, MO, USA). Prior to each biofilm assay, 1% glucose supplemented tryptic soy broth (TSB) (GSTSB) was incubated for 24 h. Brain heart infusion (BHI) broth (Difco, Detroit, MI, USA) was used for biofilm susceptibility, checkerboard, and time kill. In each assay, the BHI was supplemented with 50 mg/L calcium and 12.5 mg/L magnesium. *E. faecium* was plated for colony counts on BHI agar (Difco). BHI agar was prepared for use at 0.5 and 1.5%, dependent on assay (Oxoid, Lenexa, KS, USA). Broth used in assays containing DAP was supplemented with an additional 25 mg/L of calcium [42].

### 4.3. Bacteriophage Source and Propagation

Phages NV-497, NV-503-01, and NV-503-02 as well as phages 9181, 9183, and 9184, were isolated from wastewater treatment facilities in Maryland and Colorado and provided by the Department of the NAVY and the Duerkop laboratory, respectively [43]. ATCC 113 Phage (ATCC 19950-B1) and propagating organism *E. faecium* (ATCC 19950) were purchased from ATCC (Manassas, VA, USA). Phages were propagated in liquid culture to yield high titer stocks (≥10^9^ PFU/mL) with lysates filtered with a 0.2 µm filter to remove remaining bacteria and cell debris [31,37,44]. Filtered phage was then stored, protected from light at 2–8 °C.

### 4.4. Biofilm Quantification Assay

Biofilm production for each *E. faecium* strain was evaluated, as previously described [38,39]. In brief, *E. faecium*-inoculated BHI broth was added to each well of a microtiter plate (96-well) and placed in a 37 °C shaker incubator for 24 h for biofilm formation around the bottom ring of each well. Next, planktonic cells are washed from the wells with sterile water and stained with 0.2% crystal violet for 30 min. The plate was then washed, and biofilm dissolved with 33% glacial acetic acid. Biofilm quantification was measured at OD_560_ (OD) before and after the addition of glacial acetic acid. Samples were analyzed for production compared to wells containing media alone, which was used as a negative control (OD_c_). Classification of adherence capabilities for each strain was categorized into one of four categories: none (OD ≤ OD_c_), low (OD_c_ < OD ≤ 2xOD_c_), medium (2xOD_c_ < OD ≤ 4xOD_c_), or high (4xOD_c_ < OD) [38,39,45].

### 4.5. Phage Sensitivity Assay

Phage activity for eight *E. faecium* strains was tested using the small drop overlay method or “spot testing” on BHI plates, as previously described [44,46]. First, 100 µL of *E.* faecium planktonic 18 h overnight culture was mixed with 5 mL of 50 °C 0.5% BHI overlay. Next, the mixture was poured uniformly onto the BHI plate. Once the overlay was set (approximately 10 min after pouring), phages were spotted in 5 µL increments onto the overlay in 10-fold serial dilutions, incubated overnight at 37 °C, then counted [46]. Phage was noted to be active if clearing of the bacterial lawn was identified where phage was spotted, and discrete plaques were observed in the diluted phage spots. Plaques at specific dilutions were counted to determine the phage titer [46]. Phage susceptibility was classified based on phage titer, which was measured with plaque-forming units (PFUs). High susceptibility was indicated with >10^7^ PFU (green), medium susceptibility was indicated with 10^3^ to 10^7^ PFU (yellow), and low susceptibility with <10^3^ PFU (orange). If no PFU were identified then the phage was considered to be nonsusceptible (NS, red).

### 4.6. Antibiotic Susceptibility Testing

The pin-lid method using the Calgary Biofilm Device (CBD) was conducted in duplicate to determine minimum biofilm inhibitory concentration (MBIC) values for each *E. faecium* strain, as described previously [47,48,49]. *E. faecium*-inoculated GSTSB was added to the 96-well microtiter plate with the pin-lid placed on top of the microtiter plate then incubated at 37 °C for 24 h. The following day, the lid was removed from the microtiter plate, rinsed with phosphate buffer solution, then placed on a separate microtiter plate containing serial antibiotic dilutions according to the Clinical and Laboratory Standards Institute (CLSI) broth microdilution (BMD) method and inoculated at 37 °C for 24 h [50,51,52]. The pin-lid was removed to record the MBIC, which was defined per CLSI as the column with highest antibiotic dilution demonstrating no bacterial growth.

### 4.7. Modified Checkerboard for Antibiotic and Bacteriophage Synergy Screening

A modified checkerboard MBIC assay was used to assess PAS against *E.faecium* isolates R497 and HOU503, as previously described. In brief, 200 µL of 1% GSTSB inoculated with 5 × 10^5^ starting inoculum of test organism was distributed in 96-well round-bottom microtiter plates covered with a 96-pin lid and statically incubated for 24 h at 37 °C, allowing biofilm formation on the pins. The next day, in a separate 96-well plate, 100 µL of BHI was placed in each well, then 100 µL of a single antibiotic (DAP or AMP) at 4xMBIC was added to each well in column 1, then serially diluted 2-fold through the tray through column 9. Next, 100 µL of the other antibiotic was added to columns 1 to 10 of row one and serially diluted 2-fold from row 1 through row 7. In checkerboards where phage was added to single antibiotic (DAP or AMP), 20 µL of phage at an MOI of 10 was added to each well in columns 1 to 10 of row 1. Phage was then serially diluted 10-fold from row 1 through row 7. To assess PAS in checkerboards containing both DAP and AMP, phage was added at a constant subinhibitory MOI (based on single PAS checkerboard results) following completion of DAP and AMP dilutions. In each checkerboard, columns 11 and 12 were designated as growth control and media control, respectively. Once dilutions were complete, then pin-lid from the original tray was removed from the first 96-well plate and placed on the 96-well plate containing the checkerboard dilutions. The plate was then at 37 °C for 24 h, and then read with a spectrophotometer at OD_570_. The fractional inhibitory concentration (FIC) for each checkerboard was calculated with FIC index of ≤0.5, 1–4, and >4 indicating synergy, additivity, and antagonism, respectively [53,54].

### 4.8. Time Kill Analyses

Evaluation of bacterial growth suppression was performed with TKA over a 24 h time course to evaluate suppression of bacterial growth and PAS in microwell plates, as previously described [55,56]. First, four sterile 3-mm polyurethane beads were placed in each well followed by 2 mL total volume of *E. faecium* at 6 log_10_ CFU/mL and 1% GSTSB in 1:9 ratio. Plates were incubated for 24 h at 37 °C to yield biofilm formation on the beads. The 2 mL of inoculated broth was then aspirated from each well, without disturbing the biofilm-covered beads, and replaced with BHI broth supplemented with 250 µL of calcium chloride per 50 mL of broth. The starting inoculum on each bead for each TKA was 10^6.5–7^ CFU/mL. DAP and AMP were added to their designated wells at 0.5xMBIC and free physiological peak concentration (free peak = 72 µg/mL), respectively. Phage dosing was optimized to a subinhibitory MOI (ratio of phage to target organism) of 0.01 for each phage based on modified checkerboard MBIC results. Antibiotic was added to each well first directly followed by the addition of phage. TKA growth curves were constructed from sterilely removed beads at 0 (prior to the addition of phage and/or antibiotic), 4, 8, and 24 h. Each bead was placed in an Eppendorf tubed containing 0.9 mL of 0.9% saline and stored at 2–8 °C for 24 h to inactivate the phage. Each sample was then thawed and processed to remove biofilm with 1-min vortex and sonication (20 Hz, Bransonic 12 Branson Ultrasonic Corporation) intervals for a total of 6 min. Antibiotic carryover was eliminated from each sample with dilutions in 0.9% saline, as appropriate [57]. Diluted samples were plated on BHI agar (easySpiral, Interscience for Microbiology, Saint Nom la Breteche, France, detection limit of 10^2^ CFU/mL), and incubated at 37 °C for 24 h followed by counting of bacterial colonies (Scan 1200, Interscience for Microbiology, Saint Nom la Breteche, France). Synergy and bactericidal activity were defined as a ≥2 log_10_ CFU/mLkill compared to the most effective agent (or double-combination regimen) and a ≥3 log_10_ CFU/mL reduction from baseline at 24 h. Single drug/phage exposures in biofilm TKA included DAP, AMP and each of the four phages. Additionally, combination evaluations were performed with DAP, AMP, and DAP plus AMP with combinations of two, three, and four phages. Statistical analysis was carried out using SPSS version 21.0 (IBM Corp., Armonk, NY, USA) software. Significant differences between phage-antibiotic regimens in terms of bacterial killing metrics (i.e., extent of TKA reductions in log_10_ CFU/mL counts at time 0 vs. 24 h) was assessed by analysis of varianace (ANOVA) with Tukey’s *post hoc* test (*p* < 0.05).

### 4.9. Resistance Testing

Following the infection of *E. faecium* strains R497 and HOU503 in 24 h TKA with selected phages, modified bacteriophage insensitive mutants (BIM) testing was conducted as previously described to evaluate frequency of resistance (FOR) [41,46,58]. First, for each TKA sample, a mixture of 100 µL high titer phage and 10 µL TKA sample was incubated at 37 °C for 10 min. The mixture was then added to 5 mL of 0.5% BHI overlay and quickly poured onto square BHI plates. Colonies arising on the plate were counted following incubation at 37 °C for 24 h and again after an additional 24 h at room temperature. FOR was calculated by taking the colony count from 24 h TKA samples and dividing it by the number of colonies identified on the 24 h and 48 h plates.

The double drop method was then used to evaluate phage sensitivity in colonies surviving 24 h TKA in the following manner: first, 10 µL aliquots of high titer phage were spotted on BHI plates then 5 µL of overnight *E. faecium* culture from each BIM was spotted on top of the first 10 µL aliquot. Plates were incubated at 37 °C for 24 h with aliquot spots then compared to bacterial control spots without phage. If there was no difference between the phage-bacteria spot and the control spot the phage was considered resistant, if phage activity was identified in the bacteria spot then the phage was intermediate, and if <10 colonies were identified in the spot then the phage was sensitive [41].

## Figures and Tables

**Table 1:** Biofilm quantification (optical density (OD), compared to control) and phage susceptibility against *E. faecium* clinical isolates.

Enterococcus Faecium Clinical Isolates	DAP	VAN	Biofilm Quantification	Phage Susceptibility								
				ATCC Phage 113	NV-497	NV-503-01	NV-503-02	NV-S447-01	NV-S447-02	9181	9183	9184
**R497**	R	R	Medium									
**HOU503**	SDD	R	High									
**5938**	R	R	Low									
**S447**	SDD	R	Low									
**S80849**	SDD	R	Low									
**SF11499**	SDD	R	Low									
**SF12047**	SDD	R	Low									
**12311**	SDD	R	Low									

Phage susceptibility was classified as high, medium, or low based on plaque-forming unit (PFU) counts where >10^7^ PFU/mL was defined as high (green), 10^3^ to 10^7^ PFU/mL was defined as medium, and <10^3^ PFU/mL was defined as low. Phage was classified as non-susceptible (NS) if no PFU were identified (red). Abbreviations: DAP, daptomycin; VAN, vancomycin; R, resistant; SDD, susceptible dose-dependent.

**Table 2:** Evaluation of phage resistance in R497 and HOU503 at the end of 24 h time kill analyses. Phages are represented in the table as follows: 1, ATCC 113; 2, NV-497; 3, NV-503-01; 4, NV-503-02.

	Single Phage				2-Phage Cocktails						3-Phage Cocktails				4-Phage Cocktail
	1	2	3	4	1,2	1,3	1,4	2,3	2,4	3,4	1,2,3	1,2,4	1,3,4	2,3,4	1,2,3,4
**R497**															
DAP															
AMP															
DAP + AMP															
**HOU503**															
DAP															
AMP															
DAP + AMP															

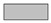
 = resistant 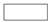 = susceptible

## Data Availability

Not applicable.

## Support Statement

This work was supported by work unit number A1417.

## Copyright Statement

Dr. Biswas is a federal employee of the United States government. This work was prepared as part of his official duties. Title 17 U.S.C. 105 provides that ‘copyright protection under this title is not available for any work of the United States Government’. Title 17 U.S.C. 101 defines a U.S. Government work as work prepared by a military service member or employee of the U.S. Government as part of that person’s official duties.
